# Fluorescence spectroelectrochemistry for the study of electrochemiluminescence of the CdTe quantum dots/tripropylamine coreactant system

**DOI:** 10.1007/s44211-026-00906-0

**Published:** 2026-04-03

**Authors:** Masayuki Nakayama, Musashi Nakajima, Atom Hamasaki, Fumiki Takahashi, Hirosuke Tatsumi, Jiye Jin

**Affiliations:** Department of Chemistry, Faculty of Science, Shinshu University, 3-1-1 Asahi, Matsumoto, Nagano 390-8621 Japan

**Keywords:** CdTe quantum dots, Electrochemiluminescence, Tripropylamine, In situ fluorescence measurement

## Abstract

This study investigates the electrochemiluminescence (ECL) behavior of water-soluble cadmium telluride quantum dots (CdTe QDs) synthesized via a microwave-assisted hydrothermal method. The ECL response was examined in the presence of tri-n-propylamine (TPrA) as a coreactant, revealing two anodic ECL emissions, denoted as ECL_1_ and ECL_2_, at potentials of + 0.85 V and + 1.2 V, corresponding to TPrA oxidation and CdTe QD oxidation, respectively. These emission intensities showed strong dependence on pH, suggesting multiple mechanistic pathways. In situ fluorescence microscopy was employed to observe interactions between electrogenerated TPrA intermediates and CdTe QDs at the electrode surface, providing insights into the reaction mechanisms. It is proposed that ECL_1_ arises from reactions involving the TPrA^•+^ intermediate under mildly basic conditions (pH 8–9), where both TPrA^•+^ and TPrA^•^ radicals facilitate electron transfer with CdTe QDs, resulting in low-potential ECL emission. The findings demonstrate that TPrA^•+^ acts as both a fluorescence quencher and a reactive intermediate, advancing understanding of the pH-dependent anodic ECL mechanism of semiconductor QDs.

**Graphical abstract:**

**Supplementary Information:**

The online version contains supplementary material available at 10.1007/s44211-026-00906-0.

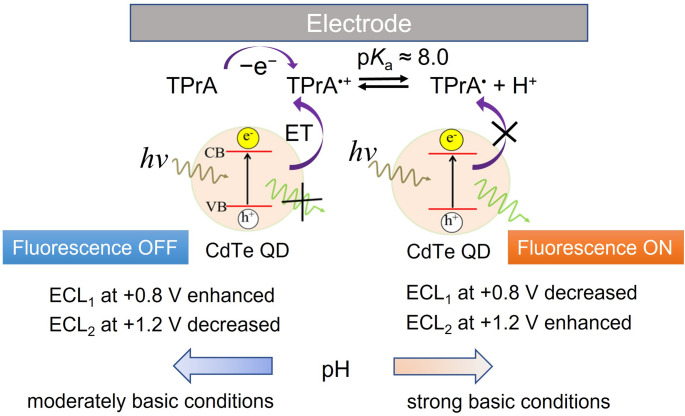

## Introduction

Electrogenerated chemiluminescence (ECL) is a light-emitting process initiated by electrochemical reactions that generate electronically excited states at electrode interfaces. Since the pioneering discovery of the [Ru(bpy)_3_]^2^⁺/tripropylamine (TPrA) system by Bard and co-workers, ECL has developed into a powerful analytical and imaging technique distinguished by its high sensitivity, wide dynamic range, and negligible optical background noise [1–3]. By combining electrochemical control with luminescence detection, ECL has been widely applied in biosensing, materials characterization, and light-emitting devices [4–9].

Semiconductor quantum dots (QDs), together with molecular luminophores, have emerged as important ECL emitters due to their size-tunable emission, narrow spectral bandwidths, and high photostability [10–13]. Their well-defined band structures enable diverse ECL pathways under both cathodic and anodic conditions. In cathodic ECL systems with persulfate (S_2_O_8_^2^⁻) as a coreactant, QDs are first reduced to radical anions (QDs^•^⁻), while S_2_O_8_^2^⁻ is reduced to sulfate radicals (SO_4_^•^⁻), which subsequently reacts with QDs^•^⁻ to generate an excited singlet state of the QDs (QDs^*^) [14, 15]. In anodic systems, TPrA is one of the most widely used coreactants. Upon oxidation at the electrode, TPrA loses an electron, forming a radical cation (TPrA^•^⁺), which rapidly deprotonates to produce a highly reducing radical (TPrA^•^) [8, 16]. In analogy to the classical [Ru(bpy)_3_]^2+^/TPrA system, TPrA^•^ is generally assumed to reduce oxidized QDs (QDs^•^⁺), producing excited states (QDs^*^) responsible for light emission [17, 18].

Although the optical and electrochemical properties of QDs are known to show size-dependent characteristics due to their wide range of band gap energies (*E*_g_), many issues remain regarding ECL mechanisms in the anodic potential range. In our previous study [17], we examined how QD particle size influences the ECL response of the cadmium telluride (CdTe) QDs/TPrA system and observed that QD-based anodic ECL often emits at potentials more positive than the formal oxidation potential of TPrA. Additionally, low-potential ECL signals are frequently seen even when direct oxidation of the QDs is thermodynamically unfavorable. This raises an important mechanistic question: does low-potential anodic ECL from QDs require the direct electrochemical oxidation of the QDs, or can it occur solely through TPrA-derived intermediates? Moreover, the specific role of the TPrA radical cation (TPrA^•^⁺), which acts as a precursor to TPrA•, remains insufficiently understood in QD systems.

Recent spectroelectrochemical studies, especially in the [Ru(bpy)_3_]^2+^/TPrA system, have shown the effectiveness of time-resolved optical techniques in directly identifying reactive intermediates and improving mechanistic understanding [19]. However, similar in situ spectroscopic validation of intermediate species in QD-based ECL systems remains scarce. In the QDs-based ECL system, where band-edge energetics, surface states, and interfacial charge-transfer kinetics differ fundamentally from molecular luminophores, direct optical probing of excited-state behavior during electrochemical polarization is particularly crucial.

In this study, we synthesized water-dispersible CdTe QDs using a microwave-assisted hydrothermal method and systematically examined their anodic ECL behavior in the presence of TPrA. By combining electrochemical analysis with in situ fluorescence spectroelectrochemistry, we directly monitored the interaction between electrogenerated TPrA intermediates and CdTe QDs during potential sweeps. This approach enables real-time correlation between fluorescence quenching and electrochemical oxidation of TPrA, thereby enabling the mechanistic separation of (i) QD oxidation processes and (ii) TPrA-mediated electron-transfer pathways.

Our results show that low-potential ECL in the CdTe QDs/TPrA system can occur near the oxidation potential of TPrA (+ 0.85 V), which strongly depends on the equilibrium between the oxidized TPrA species (TPrA^•^⁺/TPrA^•^). Under moderately basic conditions (pH 8–9), where both TPrA^•^⁺ and TPrA^•^ play essential and collaborative roles near the electrode interface. TPrA^•^⁺ is shown to function not only as a precursor to TPrA^•^ but also as an electron acceptor capable of modulating excited-state populations of the QDs. These findings provide mechanistic clarification of anodic QD-based ECL and establish fluorescence spectroelectrochemistry as a powerful framework for probing intermediate species in semiconductor nanocrystal systems.

## Experimental section

### Materials and reagents

Cadmium chloride (CdCl_2_), 3-mercaptopropionic acid (MPA), and tris(hydroxymethyl)aminomethane (Tris) were purchased from Nacalai Tesque (Kyoto, Japan). Sodium tellurite pentahydrate (Na_2_TeO_3_·5H_2_O) and sodium hydroxide (NaOH) were obtained from Kanto Chemical Co., Inc. (Tokyo, Japan). Tri-n-propylamine (TPrA), 2-propanol, and hydrochloric acid (HCl) were supplied by Wako Pure Chemical Industries, Ltd. (Osaka, Japan). Tris buffer solutions were prepared from a Tris stock solution, and the pH was adjusted with 0.1 M HCl or 0.1 M NaOH. Ultrapure water used throughout the experiments was produced with a WS-200 purification system (Yamato Scientific Co., Ltd., Tokyo, Japan).

### Apparatus

ECL measurements were conducted at room temperature using a CV-27 potentiostat/galvanostat (BAS Japan) in a standard three-electrode setup. A glassy carbon disk electrode (3 mm diameter; Tokai Carbon Co., Tokyo, Japan) served as the working electrode, with a platinum wire as the counter electrode and an Ag/AgCl reference electrode (RE-1, BAS Japan). Before each measurement, the working electrode was mechanically polished with a 0.3 μm alumina slurry, sonicated in an ultrasonic bath, and rinsed thoroughly with ultrapure water to ensure a clean, reproducible surface.

**Fig. 1:**
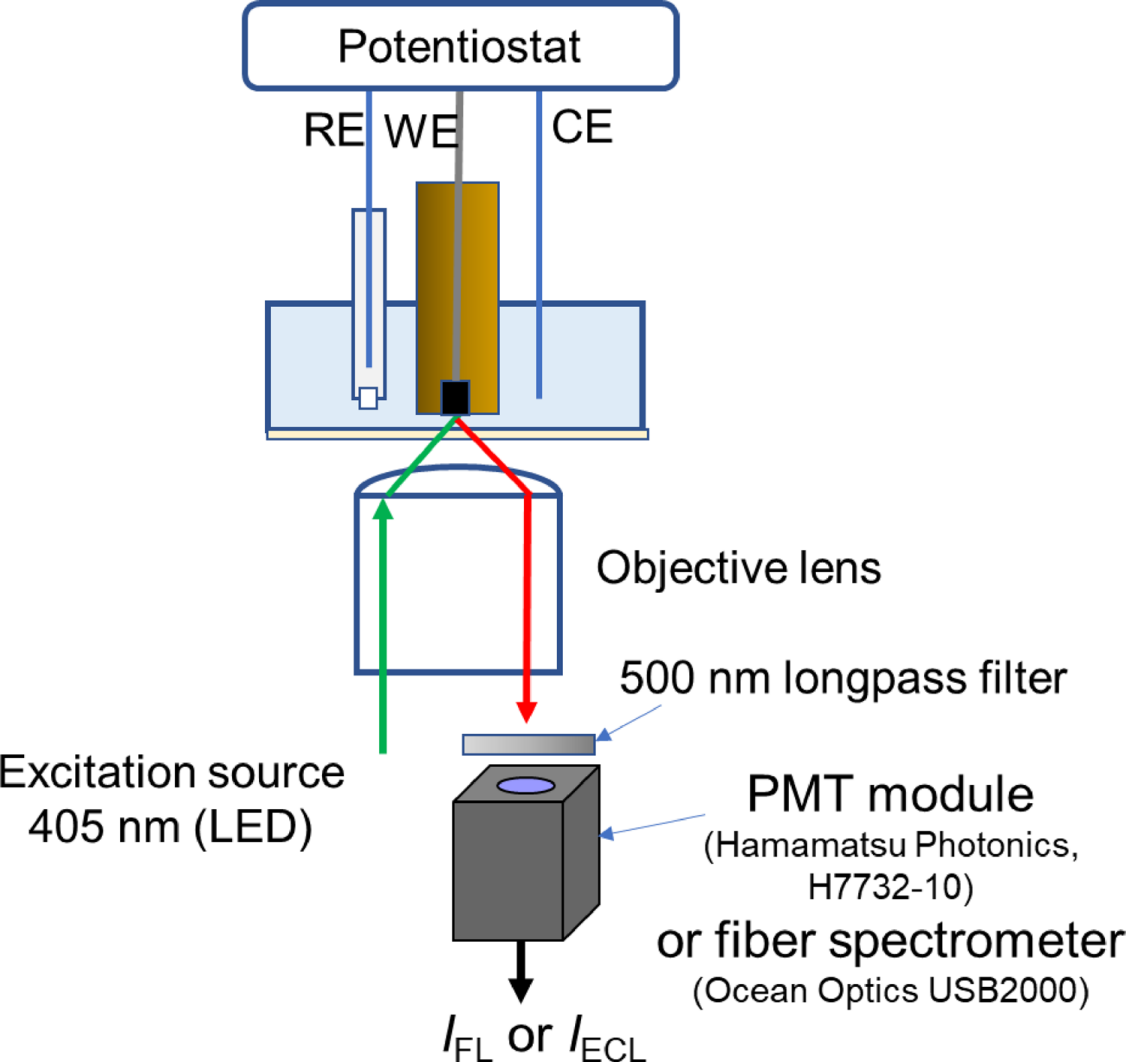
Schematic illustration of the experimental setup for detecting fluorescence (*I*_FL_) and electrochemiluminescence (*I*_ECL_₎ signals from the electrode surface using a microscope-based spectroelectrochemical system

A custom-made spectroelectrochemical cell with an optical window was used to measure fluorescence and ECL signals (Fig. 1). Fluorescence signal (*I*_FL_) from the electrode surface was observed using an inverted optical microscope (CKX53, Olympus, Japan) equipped with a 20 × objective lens. Excitation at 405 nm was provided by a fiber-coupled LED source (FOLS-01; Sawaki Kobo Co., Ltd., Japan). The 405 nm LED light was reflected by a dichroic mirror, passed through a set of scanning mirrors, and then focused by an objective lens onto the electrode surface. Fluorescence signals were collected and filtered by a dichroic mirror. The signals were detected using a Hamamatsu H7732-10 photomultiplier tube (PMT) module with a 500 nm long-pass optical filter or a miniature spectrometer (USB2000; Ocean Optics, USA) via an optical fiber. The system allows detection of ECL emission (*I*_ECL_) without realignment by switching off the excitation light, enabling direct comparison of fluorescence and ECL signals from the same surface region of the electrode.

### Synthesis of CdTe QDs

CdTe QDs were synthesized through a microwave-assisted hydrothermal method adapted from a previously reported procedure [17]. A microwave chemical reactor (Green Motif I) from J. Science Lab Co., Ltd. (Kyoto, Japan), equipped with a controllable temperature unit, was used to synthesize CdTe QDs. Cadmium chloride and sodium tellurite served as Cd and Te precursors, respectively, and MPA was used as a stabilizing and capping ligand. After dissolving the precursors in water, the solution was adjusted to approximately pH 9.0 with 0.1 M NaOH. The mixture was then subjected to microwave irradiation under sealed conditions, promoting controlled nucleation and growth of CdTe nanocrystals. The resulting MPA-capped CdTe QDs were purified by precipitation with 2-propanol and then redissolved in 0.1 M Tris buffer (pH 8.5).

## Results and discussion

### Electrochemistry and ECL properties of the CdTe QD/TPrA system

**Fig. 2:**
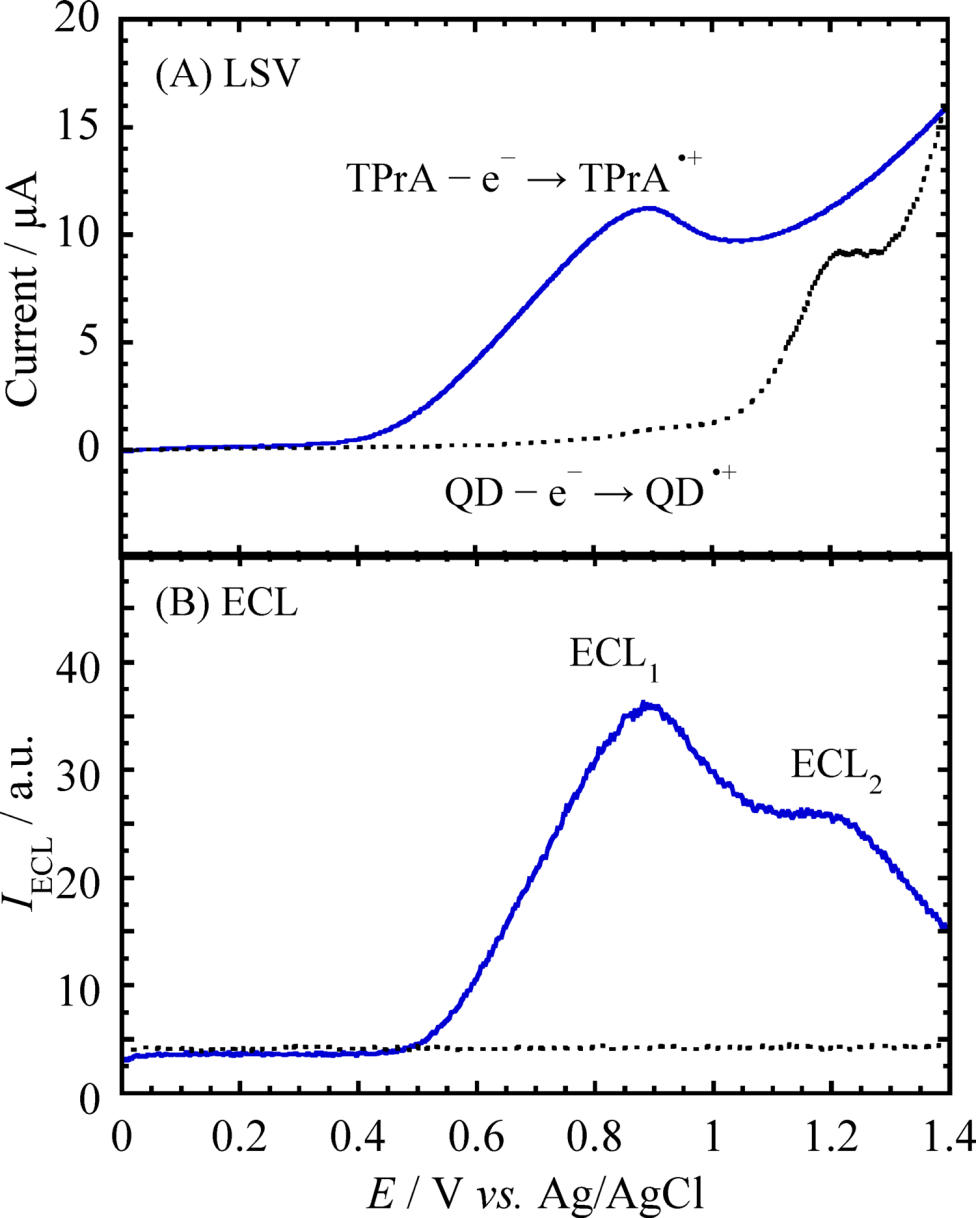
**A** Cyclic voltammograms of 1 μM CdTe QDs (*D* = 3.2 nm) and 1 mM TPrA in Tris buffer solution (pH 8.5), respectively. **B** ECL responses of CdTe QDs (1 μM, *D* = 3.2 nm) in Tris buffer solution (pH 8.5) containing 1 mM TPrA. The dotted line represents the background responses obtained in the absence of TPrA

The electrochemical and optical properties of the CdTe QDs were characterized by cyclic voltammetry, UV–Vis absorption spectroscopy, and fluorescence spectroscopy, confirming a high quantum yield and a narrow emission profile. The purified CdTe QDs exhibited strong fluorescence at 595 nm, characteristic of CdTe nanocrystals with an intermediate particle size. Particle size and bandgap energy (*E*_g_) were estimated from UV–Vis absorption spectra and cyclic voltammograms (Figs. 1S and 2S). The first excitonic absorption peak indicated an average particle size of about 3.2 nm, and the onset wavelength (*λ*_onset_) yielded an *E*_g_ of 2.0 eV, as calculated using the method of Fendler et al. [20].

The electrochemistry and ECL of the CdTe QDs/TPrA system at a glassy carbon electrode were investigated. The dotted line in Fig. 2A shows the linear sweep voltammogram of 1 μM CdTe QDs (*D* = 3.2 nm) dispersed in Tris buffer (pH 8.5). An oxidation wave appears at approximately + 1.2 V vs. Ag/AgCl, attributed to the electrochemical oxidation of CdTe QDs via QD – e⁻ → QD^•^⁺, indicating formation of the radical cation species (CdTe QD^•^⁺) [21, 22]. The solid line in Fig. 2A corresponds to the LSV of 1 mM TPrA, which exhibits an oxidation wave at approximately + 0.85 V vs. Ag/AgCl. This feature is ascribed to the one-electron oxidation of TPrA at the electrode surface, yielding the radical cation intermediate [TPrA^•^⁺ = (CH₃CH₂CH₂)₃N^•^⁺] [23].

Figure 2B shows the corresponding ECL responses of 1 μM CdTe QDs in Tris buffer. To stabilize the electrode surface, *I*_ECL_ was measured after 5 successive cyclic voltammetry (CV) scans over a potential range from 0 to + 1.4 V vs. Ag/AgCl. No detectable ECL emission was observed in the solution containing CdTe QDs alone (dotted line). However, upon addition of 1 mM TPrA, two distinct ECL signals, denoted ECL_1_ and ECL_2_, appeared near the oxidation potentials of TPrA (+ 0.8 V) and CdTe QDs (+ 1.2 V), respectively.

Based on previous studies [17, 24], ECL_2_ could be attributed to the classical “oxidative–reduction” coreactant mechanism. The radical cation TPrA^•^⁺ readily undergoes deprotonation under mildly basic conditions to produce the highly reducing neutral radical [TPrA^•^ = (CH₃CH₂)₂C^•^HCH₂CH₃], which has a formal potential of *E*°’ ≈ − 1.7 V vs. Ag/AgCl [25]. When CdTe QDs are electrochemically oxidized, holes are generated in the valence band (VB), resulting in the formation of CdTe QD^•^⁺. The neutral radical TPrA^•^ subsequently acts as a substantial electron donor. It injects an electron into the excited energy level (*E*ₛ) of the oxidized QDs, leading to the formation of the excited-state species CdTe QD^*^, as represented by Eq. (1).

1 $${\mathrm{QD}}^{ \bullet + } + {\mathrm{TPrA}}^{ \bullet } \to {\text{ QD}}^{*} + {\mathrm{Products}}$$

However, this mechanism failed to explain the ECL_1_ emission, which occurs at potentials lower than the oxidation potential of CdTe QDs. Therefore, it is suggested that ECL_1_ proceeds via a different reaction pathway. At the potential corresponding to ECL_1_, TPrA is oxidized to the cation radical TPrA^•^⁺, implying that the ECL_1_ signal arises from a reaction pathway involving TPrA^•^⁺.

### Effect of pH on ECL of the CdTe QD/TPrA system

**Fig. 3:**
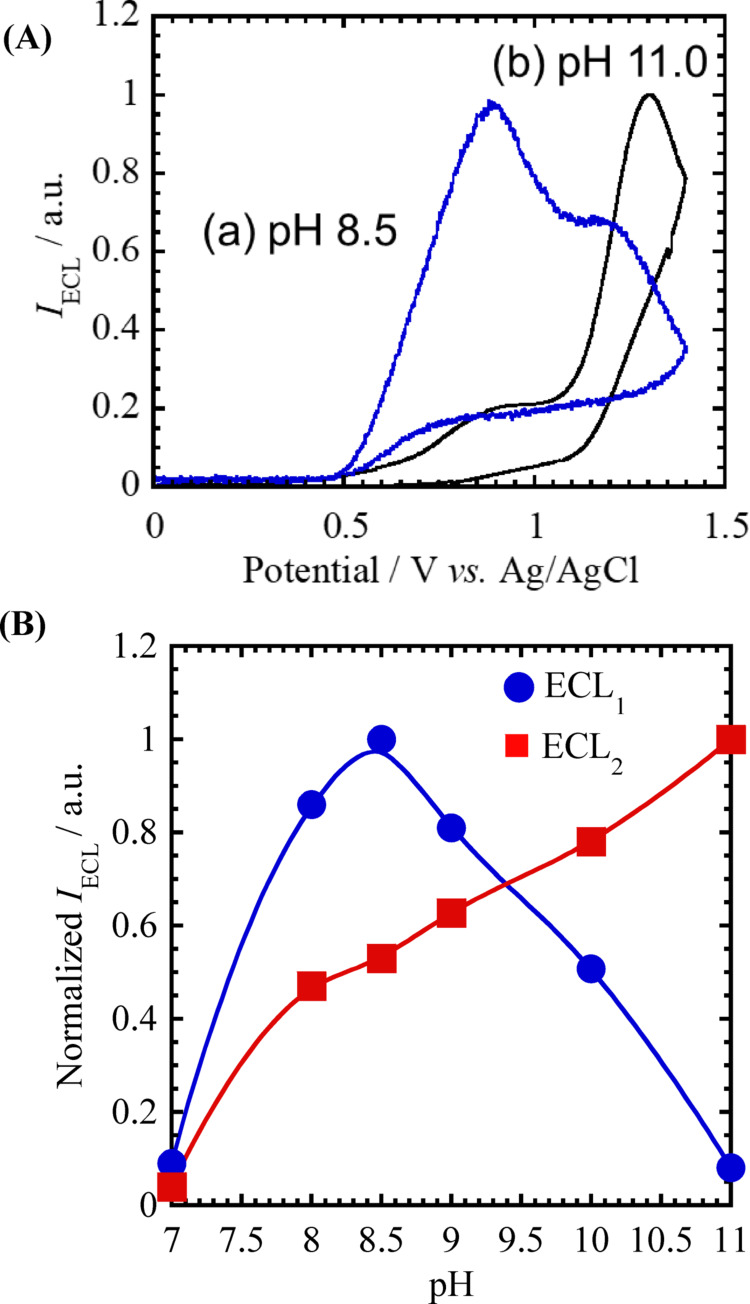
**A** ECL responses of 1 μM CdTe QDs and 1 mM TPrA in Tris buffer solutions with pH values of **a** 8.5 and **b** 11.0. The potential scan rate was 50 mV s⁻^1^. **B** Effect of pH on the relative ECL intensities of ECL_1_ (●) and ECL_2_ (■). ECL_1_ and ECL_2_ were normalized independently to their respective maximum intensities. The ECL measurements were performed under the same conditions as in (**A**), and the pH was adjusted by adding small amounts of NaOH or HCl

Previous studies have demonstrated that the stability and reactivity of the TPrA^•^⁺ radical cation are strongly influenced by solution pH [8, 26]. To better understand this effect, cyclic voltammetry and ECL measurements were performed on the CdTe QDs/TPrA system in Tris buffer solutions with various pH values. Figure 3A displays the ECL responses observed at pH 8.5 and 11. When the pH was raised to 11, the low-potential emission (ECL_1_) significantly decreased, leaving only the high-potential emission (ECL_2_) at + 1.2 V vs. Ag/AgCl. This behavior suggests that the formation and stability of radical intermediates at the electrode surface are highly sensitive to pH.

The dependence of ECL_1_ and ECL_2_ intensities on pH is summarized in Fig. 3B. On the vertical axis, ECL_1_ and ECL_2_ were normalized separately to their respective maximum peak intensities to facilitate comparison of their pH dependence. As pH increases, ECL_1_ intensity reaches a clear maximum at pH 8–9, whereas ECL_2_ intensity continues to increase at higher pH values. When pH is below 7, no detectable ECL response (for both ECL_1_ and ECL_2_) was observed. The initial step in the anodic ECL mechanism involves the one-electron oxidation of TPrA at the electrode surface, producing the radical cation TPrA^•^⁺, which quickly deprotonates at the α-carbon. This deprotonation is governed by an equilibrium with an estimated p*K*ₐ of approximately 8.0 [27].

2 $${\mathrm{TPrA}}^{ \bullet + } \rightleftarrows {\mathrm{TPrA}}^{ \bullet } + {\text{ H}}^{ + }$$

At higher pH, deprotonation of TPrA^•^⁺ becomes more favorable, producing the highly reducing neutral radical TPrA^•^. This species enhances electron transfer to QDs^•^⁺, thereby promoting the ECL_2_ process at + 1.2 V vs. Ag/AgCl.

In contrast, the intensity of ECL_1_ reaches maximum under moderately basic conditions (pH 8–9), where both TPrA^•^⁺ and TPrA^•^ coexist at the electrode surface in a defined equilibrium ratio. The concentration profiles of TPrA^•^⁺ and TPrA^•^ are calculated as shown in Fig. 4S in the Supporting Information, at both + 0.85 V and under these conditions. Under strongly basic conditions, rapid deprotonation diminishes the lifetime and concentration of TPrA^•^⁺, thereby inhibiting its role in the generation of excited CdTe quantum dots (QDs^*^). These observations strongly suggest that the transient TPrA^•^⁺ species is essential for ECL_1_ emission, whereas the fully deprotonated TPrA^•^ predominantly facilitates the ECL_2_ pathway.

### In situ fluorescence measurements of the CdTe QD/TPrA system

Spectroelectrochemical techniques have provided valuable optical insights into the nature and dynamics of species generated during electrochemical reactions [19, 28, 29]. In this study, the fluorescence spectra of CdTe quantum dots (QDs) measured at the electrode surface and in the bulk solution were almost identical, confirming that the emitted light originates from CdTe QDs adsorbed or diffusing near the electrode interface. To directly investigate the presence and stability of the TPrA^•^⁺ intermediate at the electrode surface, in situ spectroelectrochemical fluorescence measurements were performed using a microscope-based setup shown in Fig. 1. The fluorescence signal (*I*_FL_) was recorded under excitation at 405 nm, while the ECL signal (*I*_ECL_) was obtained by turning off the excitation source. This setup allows for direct comparison of FL and ECL signals from the same electrode region. Both ECL and FL spectra measured at the electrode surface are displayed in Fig. S3 of the Supplementary Information. The ECL emission maximum is observed at around 595 nm, which closely matches the PL spectrum of CdTe QDs, indicating that both emissions originate from the same excited state. Due to the significantly lower *I*_ECL_ intensity compared to the *I*_FL_ intensity, the contribution of ECL emission to the FL measurements can be considered negligible.

**Fig. 4:**
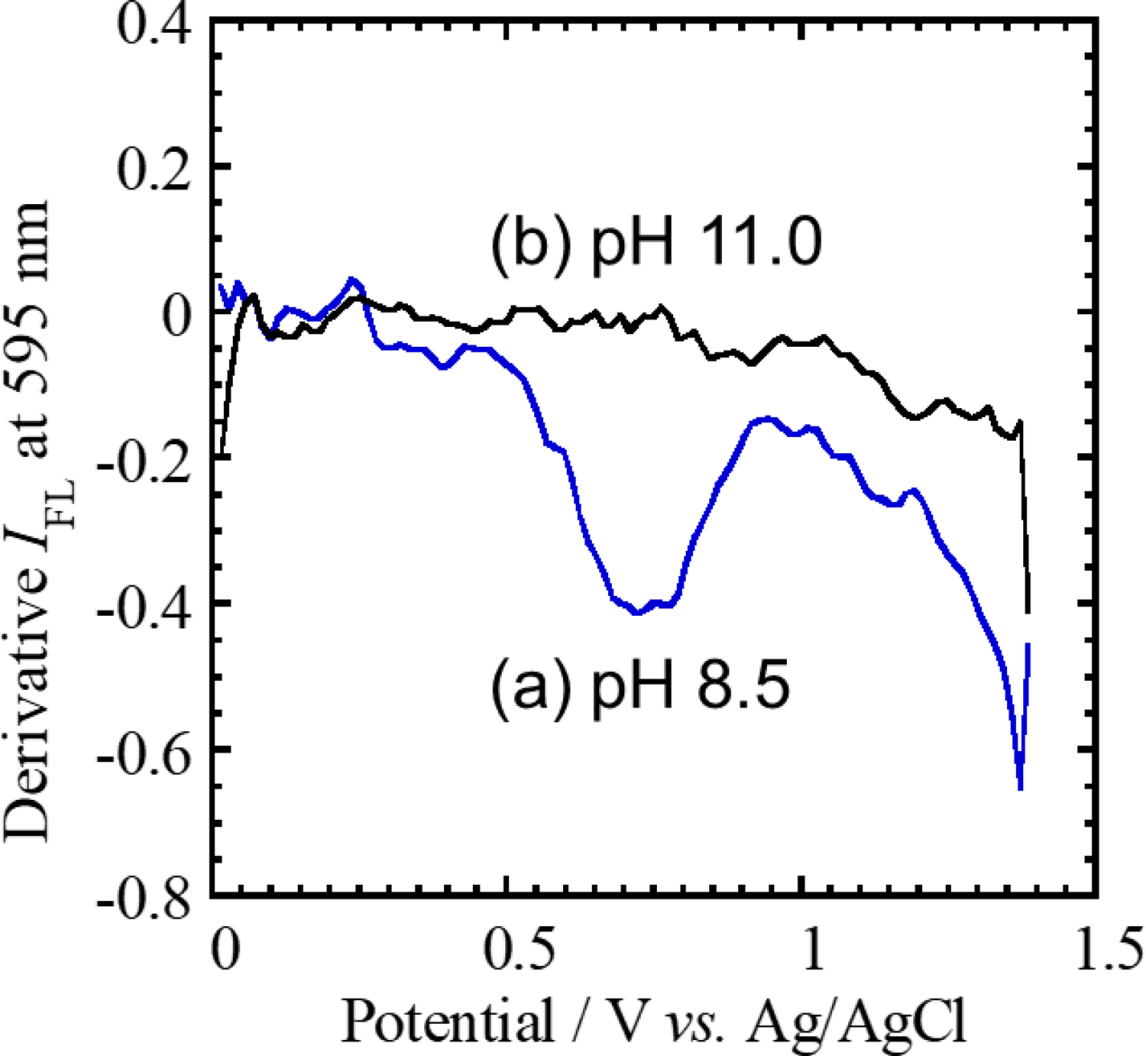
Derivative voltafluorograms recorded at 595 nm from the surface of a glassy carbon (GC) electrode. Measurements were performed in Tris buffer solutions containing CdTe QDs (1 μM) and TPrA (1 mM) at pH values of **a** 8.5 and **b** 11.0. The potential scan rate was 50 mV s⁻^1^

The variation of *I*_FL_ at 595 nm as a function of the applied potential (voltafluorogram) was recorded during potential sweeps, and the first derivative of the voltafluorogram was used to emphasize subtle changes in fluorescence intensity. As depicted in Fig. 4, when the applied potential exceeded + 0.8 V, corresponding to the oxidation of TPrA, a significant decrease in *I*_FL_ was observed at pH 8.5 (Fig. 4a). This fluorescence quenching is consistent with the transient formation of the electron-accepting species TPrA^•^⁺ at the electrode interface. In contrast, at pH 11, no significant fluorescence quenching was detected (Fig. 4b). Further decrease in *I*_FL_ occurred at more positive potentials, where CdTe QDs underwent oxidation to form CdTe QDs^•^⁺, even in a TPrA-free solution. The electron-transfer mechanisms responsible for this fluorescence quenching in the CdTe/TPrA ECL system are schematically summarized in Fig. 5S of the Supplementary Information. The fluorescence decrease near + 0.8 V is attributed to electron transfer from photoexcited CdTe QDs (CdTe QDs^*^) to TPrA^•^⁺, a process that is thermodynamically favorable. These results suggest that the formation of ECL_1_ is governed by a pH-dependent equilibrium among oxidized TPrA species, as supported by the fluorescence-quenching behavior.

**Fig. 5:**
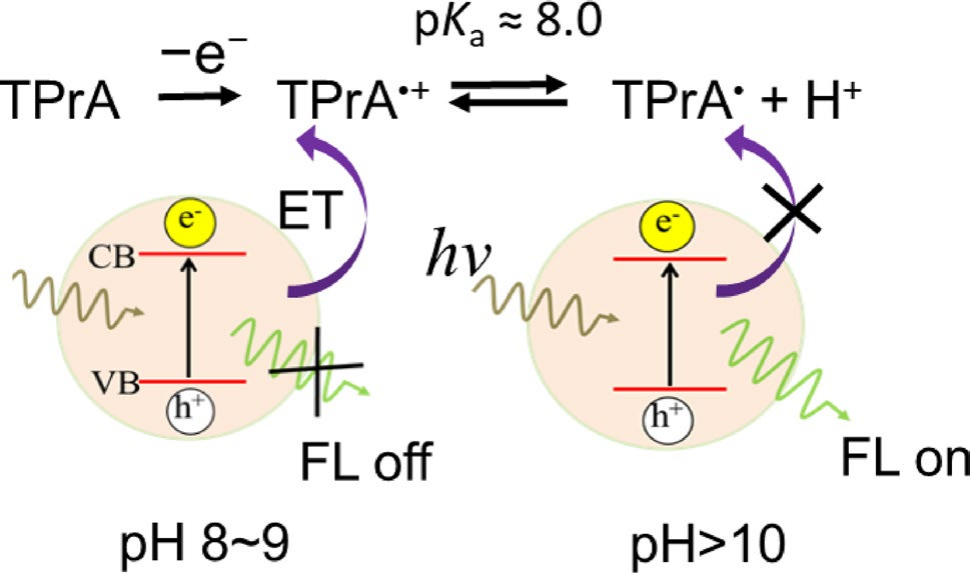
Schematic illustration of the fluorescence quenching process of CdTe QDs via electron transfer (ET) to TPrA^•^⁺ species generated at the electrode surface, highlighting the pH-dependent equilibrium between the oxidized TPrA species (TPrA^•^⁺/TPrA^•^)

The reversible fluorescence on/off response of CdTe QDs to pH variations reflects the dynamic equilibrium among oxidized TPrA intermediates, as schematically depicted in Fig. 5. Under mildly basic conditions (pH 8–9), the surface concentration of the transient radical cation TPrA^•^⁺ is sufficiently high so that it could serve as an efficient electron acceptor from photoexcited CdTe QDs^*^, resulting in fluorescence quenching (FL off). At higher pH levels, rapid deprotonation of TPrA^•^⁺ produces the neutral radical TPrA^•^, which is incapable of accepting electrons from CdTe QDs^*^, thus restoring fluorescence (FL on). The observations provided direct spectroscopic evidence for the dual role of TPrA^•^⁺ in the anodic ECL mechanism: acting both as a transient oxidant responsible for ECL_1_ emission and as a quencher of QD fluorescence.

### Proposed ECL mechanism of the CdTe QDs/TPrA system

Based on the discussions above, it is proposed that both the oxidized species (TPrA^•^⁺) and deprotonated radical (TPrA^•^) coexist in the vicinity of the electrode interface, thereby facilitating efficient interfacial electron transfer with CdTe QDs. This interaction gives rise to the low-potential electrochemiluminescence (ECL_1_) emission. The highly reducing TPrA^•^ radical, possessing an *E*°’ of approximately − 1.7 V vs. Ag/AgCl can further donate an electron to CdTe QD, resulting in the formation of the radical anion CdTe QD^•^⁻. Given that the formal potential for the redox couple CdTe QD/CdTe QD^•^⁻ is estimated to be *E*°′_CdTe/CdTe•⁻_ =  − 1.45 V vs. Ag/AgCl (refer to Fig. 2S in the Supplementary Information), reduction of CdTe QDs by TPrA^•^ is thermodynamically feasible. The TPrA^•^⁺ radical cation is continuously produced at the electrode surface and can subsequently react with CdTe QD^•^⁻ through an electron-transfer process, resulting in the formation of the excited-state CdTe QD^*^ and emitting the ECL_1_ signal. The proposed reaction pathway for ECL_1_ is summarized as follows:

3 $${\mathrm{QD}} + {\mathrm{TPrA}}^{ \bullet } \to {\mathrm{QD}}^{ \bullet - } + {\mathrm{TPrA}}$$

4 $${\mathrm{QD}}^{ \bullet - } + {\mathrm{TPrA}}^{ \bullet + } \to {\mathrm{QD}}^{*} + {\mathrm{Product}}$$

The enthalpy of annihilation (Δ*H*, in eV) in Eq. (4) can be estimated from the formal potentials of CdTe QDs^•−^ (*E*°′_CdTe/CdTe•-_ = − 1.45 V vs. Ag/AgCl) and TPrA^•+^ (*E*°′_TPrA•+/TPrA_ =  + 0.85 V vs. Ag/AgCl), using the following expression [1]:

5 $$- \Delta H = \left( {E^{{ \circ{\prime}}}_{{{\mathrm{TPrA}}^{ \cdot } + /{\mathrm{TPrA}}}} {-}E^{{ \circ{\prime}}}_{{{\mathrm{CdTe}}/{\mathrm{CdTe}}^{ \cdot } {-}}} } \right) - T\Delta S\left( {{\mathrm{eV}}} \right)$$

**Fig. 6:**
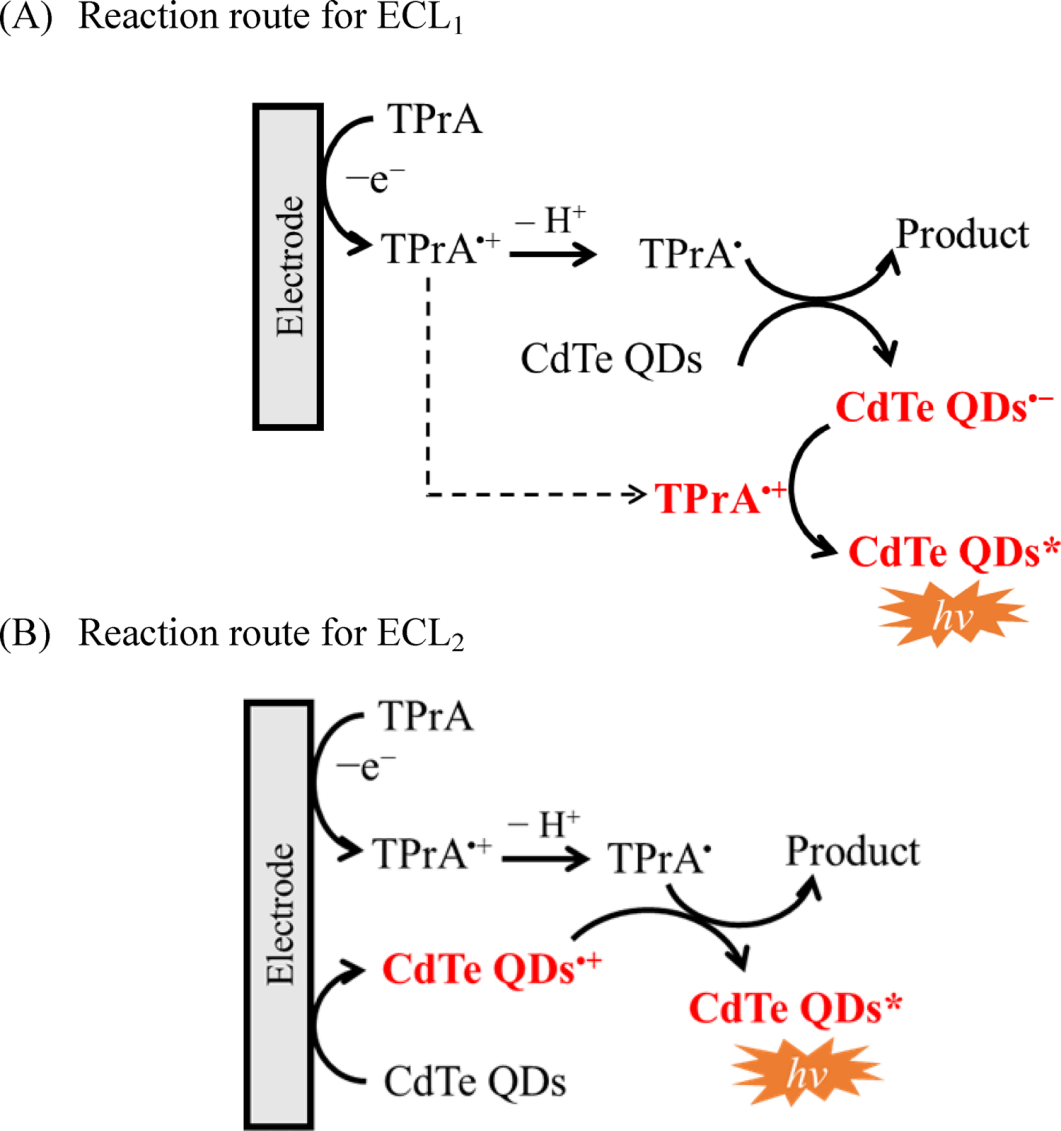
ECL mechanism for CdTe QDs / TPrA system elucidated in this study. **A** The scheme for ECL_1_ at + 0.85 V vs. Ag/AgCl. (pH 9). **B** The scheme for ECL_2_ at + 1.2 V vs. Ag/AgCl

In Eq. (5), *T*Δ*S* is the temperature-entropy change term. Its contribution at room temperature is commonly estimated to be about 0.1 eV or less [1]. This is small relative to the total enthalpy of the reaction in Eq. (4). So, − Δ*H* in Eq. (5) was calculated to be 2.2 eV, which exceeds the exciton energy level (*E*_s_ = 2.08 eV) of the CdTe QDs, indicating that the ECL_1_ process is thermodynamically feasible under moderately basic conditions (pH 8–9). The proposed mechanistic routes for ECL_1_ and ECL_2_ are summarized schematically in Fig. 6.

## Conclusions

Two distinct anodic ECL processes were identified in the CdTe QDs/TPrA system. The high-potential emission (ECL_2_), observed at approximately + 1.2 V vs. Ag/AgCl, was attributed to the direct electrochemical oxidation of CdTe QDs, followed by radiative electron–hole recombination within the generated excited-state species (CdTe QDs^*^). Conversely, the low-potential emission (ECL_1_), occurring near the oxidation potential of TPrA (+ 0.85 V), depends strongly on the equilibrium between the oxidized TPrA species (TPrA^•^⁺/TPrA^•^). It is proposed that ECL_1_ originated from interfacial reactions between CdTe QDs and transient oxidized species of TPrA, while ECL_2_ arises from direct oxidation of the QDs. In situ fluorescence measurements provided compelling evidence that TPrA^•^⁺ functions as both a fluorescence quencher and a reactive intermediate, thereby enhancing understanding of the pH-dependent anodic ECL mechanism of semiconductor QDs.

## Supplementary Information

Below is the link to the electronic supplementary material.

Supplementary Material 1

## Data Availability

The data that support the findings of this study are available from the corresponding author upon reason able request. The authors declare no competing interests.

## References

[1] A.J. Bard, *Electrogenerated chemiluminescence* (CRC Press, 2004). 10.1201/9780203027011

[2] W. Miao, J.-P. Choi, A.J. Bard, J. Am. Chem. Soc. **124**, 14478 (2002). 10.1021/ja027532v 12452725 10.1021/ja027532v

[3] K. Ino et al., Anal. Sci. **41**, 557 (2025). 10.1007/s44211-025-00723-x 39918697 10.1007/s44211-025-00723-x

[4] W. Miao, A.J. Bard, Anal. Chem. **76**, 5379 (2004). 10.1021/ac0495236 15362895 10.1021/ac0495236

[5] Y.X. Wei, H.L. Qi, C.X. Zhang, Chem. Commun. **59**, 3507 (2023). 10.1039/d2cc06930j 10.1039/d2cc06930j 36820650

[6] Y. Zhao et al., Acc. Chem. Res. **57**, 2144 (2024). 10.1021/acs.accounts.4c00273 39014959 10.1021/acs.accounts.4c00273

[7] X. Ying et al., Sens. Diagn. **2**, 480 (2023). 10.1039/D2SD00232A

[8] A. Zanut et al., Nat. Commun. **11**, 2668 (2020). 10.1038/s41467-020-16476-2 32472057 10.1038/s41467-020-16476-2 PMC7260178

[9] S.H. Kong et al., ACS Photonics **5**, 267 (2018). 10.1021/acsphotonics.7b00864

[10] Y. Zhang et al., Anal. Chem. **96**, 13690 (2024). 10.1021/acs.analchem.4c02674 39108033 10.1021/acs.analchem.4c02674

[11] F. Yin et al., TrAC Trends Anal. Chem. **169**, 117376 (2023). 10.1016/j.trac.2023.117376

[12] X.-Y. Wang et al., Talanta Open **5**, 100088 (2022). 10.1016/j.talo.2022.100088

[13] S.-N. Ding et al., Electrochem. Commun. **55**, 30 (2015). 10.1016/j.elecom.2015.03.011

[14] A.J. Stewart, K. Brown, L. Dennany, Anal. Chem. **90**, 12944 (2018). 10.1021/acs.analchem.8b03572 30280562 10.1021/acs.analchem.8b03572

[15] Y. Yan et al., J. Electroanal. Chem. **775**, 1 (2016). 10.1016/j.jelechem.2016.05.010

[16] Z. Chen, Y. Zu, J. Phys. Chem. C **112**, 16663 (2008). 10.1021/jp802873e

[17] M. Nakayama et al., Anal. Sci. **36**, 859 (2020). 10.2116/analsci.19P489 32009024 10.2116/analsci.19P489

[18] S. Tian et al., J. Electroanal. Chem. **978**, 118897 (2025). 10.1016/j.jelechem.2024.118897

[19] A.S. Danis et al., Anal. Chem. **90**, 7377 (2018). 10.1021/acs.analchem.8b00773 29756773 10.1021/acs.analchem.8b00773

[20] Y. Tian et al., J. Phys. Chem. **100**, 8927 (1996). 10.1021/jp951965l

[21] X. Liu et al., Anal. Chem. **79**, 8055 (2007). 10.1021/ac070927i 17910416 10.1021/ac070927i

[22] L. Zhang et al., J. Phys. Chem. C **112**, 4451 (2008). 10.1021/jp7097944

[23] K. Liang et al., Chem. Sci. **14**, 4152 (2023). 10.1039/D3SC00527E 37063790 10.1039/d3sc00527e PMC10094091

[24] H. Sun, P. Zhou, B. Su, Biosensors **13**, 708 (2023). 10.3390/bios13070708 37504107 10.3390/bios13070708 PMC10377090

[25] R.Y. Lai, A.J. Bard, J. Phys. Chem. A **107**, 3335 (2003). 10.1021/jp026743j

[26] P. Pastore, D. Badocco, F. Zanon, Electrochim. Acta **51**, 5394 (2006). 10.1016/j.electacta.2006.02.009

[27] S. Das, C. von Sonntag, Z. Naturforsch. B **41**, 505 (1986). 10.1515/znb-1986-0418

[28] C.M. Hill, D.A. Clayton, S. Pan, Phys. Chem. Chem. Phys. **15**, 20797 (2013). 10.1039/C3CP52756E 24196825 10.1039/c3cp52756e

[29] L. Jin et al., J. Phys. Chem. C **114**, 803 (2010). 10.1021/jp908574z

