# Supplementary material for: Fluorescence spectroelectrochemistry for the study of electrochemiluminescence of the CdTe quantum dots/tripropylamine coreactant system
Source: Anal Sci. 2026 Apr 3;42(6):431–9. doi: 10.1007/s44211-026-00906-0 (PMC13201333; doi:10.1007/s44211-026-00906-0)
Supplement: Supplementary file 1 — Supplementary Material 1 [file 44211_2026_906_MOESM1_ESM.docx]

*Supplementary Information for*

**Fluorescence Spectroelectrochemistry for the Study of Electrochemiluminescence of the CdTe Quantum Dots /Tripropylamine Coreactant System**

Masayuki NAKAYAMA^1^, Musashi NAKAJIMA^1^, Atom HAMASAKI^1^, Fumiki TAKAHASHI^1^, Hirosuke TATSUMI^1^, Jiye JIN^1^†

^1^ Department of Chemistry, Faculty of Science, Shinshu University, 3-1-1 Asahi, Matsumoto, Nagano 390-8621, Japan

Contents

1. Optical and Electrochemical Properties of CdTe QDs
2. Cyclic Voltammetry of TPrA and Digital Simulations
3. ECL emission Spectrum of CdTe QDs/TPrA System
4. Energy level diagram of CdTe QDs with a diameter of 3.2 nm

**1．Optical and Electrochemical Properties of CdTe QDs**

The resulting CdTe QDs were characterized using UV–Vis absorption and photoluminescent spectroscopic (PL) studies, as well as cyclic voltammetry. Fig. 1S presents typical absorption and PL spectra of CdTe QDs aqueous solution. The distinct absorption (A) and PL (B) peaks indicate a highly monodisperse sample. The absorption shoulder of CdTe QDs is located at 560 nm, while the emission spectrum displays an emission maximum around 595 nm upon excitation at 360 nm. According to previous reports, the size of CdTe QDs is estimated to be approximately 3.2 nm, as determined by the following empirical formula [1].

*D*(nm) = (9.8127×10^−7^) λ^3^ − (1.7147×10^−3^)λ^2^ + 1.0064λ − 194.84 (1)

In eq (1), *D* (nm) is the diameter of QD and λ (nm) is the wavelength of the first excitonic absorption peak of the UV–Vis absorption spectra. The molar extinction coefficient (ε) of CdTe QDs solution can be obtained from an empirical function of the nanocrystal size as follows [1].

*ε* (M^−1^ cm^−1^) = 10043(*d*)^2.12^ (2)

CdTe QDs have discrete energy levels and are expected to undergo electron transfer, mediated through the valence band (VB) edge and the conduction band (CB) edge. *E*_g,_ basically corresponds to the transition gap between VB and CB energy states, while PL or ECL emission originates from the excitonic transition due to radiative recombination of the electron in an excited energy level *E*_s_ with the hole in the VB. From the results of PL and ECL measurements, we believe that the electron in the lowest excited state *E*_s_ is located a little below the bottom of the CB in CdTe QDs. The *E*_s_, as measured by PL emission, may deviate from the VB–CB transition energy (*E*_g_) due to impurity levels or QD surface effect. In a case of QDs with a diameter of 3.2 nm, *E*_s_ was determined as 2.08 eV from the maximum emission wavelength in the PL spectrum.


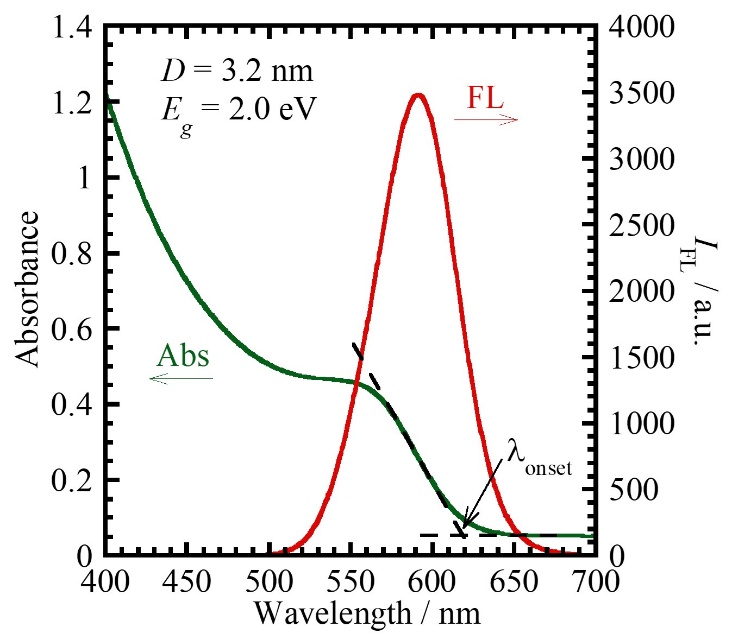


**Fig. 1S.** UV-Vis absorption and PL spectra of CdTe QDs with a diameter of 3.2 nm. PL spectrum was recorded with excitation at 360 nm.

As mentioned in the main text, the oxidation peak potential (*E*_pa_) was observed at +1.23 V *vs*. Ag/AgCl, and the formal potential of the radical cation couple, *E*º’_QDs•+/QDs_, was estimated as +1.20 V using *E*º’ ≈ *E*_pa_ − 0.029 V. Fig. S2 shows a cyclic voltammogram recorded at a GC electrode in Tris buffer (pH 8.5) containing 1 μM CdTe QDs (*D* = 3.2 nm) at a scan rate of 100 mV s^−1^. In the negative potential region, the electrochemical reduction of the CdTe QDs was observed at potentials close to −1.48 V *vs*. Ag/AgCl, which is attributed to the QD + e^−^ = QD^•–^. The formal potential of the radical anion *E*º’_QDs/QDs•–_ was estimated as −1.45 (≈ *E*_pc_+ 0.029 V), assuming the reduction process was a one-electron reaction. An anodic peak at −0.9 V during the reverse scan is most likely associated with the anodic stripping of Cd.


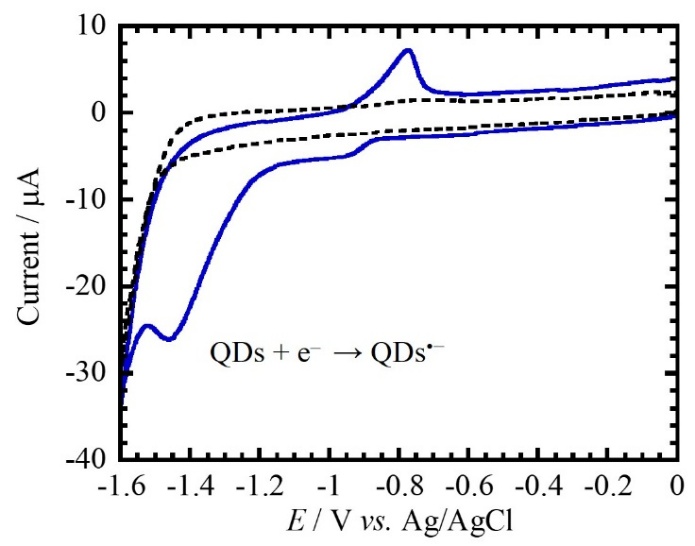


**Fig. 2S.** Cyclic voltammograms recorded on GC electrode in the (a) presence and (b) absence of 1 μM CdTe QDs in Tris buffer solution (pH 8.5). The scan rate was 100 mV/s.

**2． Cyclic Voltammetry of TPrA and Digital Simulations**

From the pH dependence of ECL observed in the CeTe QDs/TPrA system, it is suggested that the ECL at lower oxidation potential is governed by two intermediate species generated during the oxidation of TPrA, namely TPrA^•+^ and TPrA^•^. Figure 3S(A) shows the cyclic voltammogram (solid line) of 1 mM TPrA measured at a GC electrode in 0.1 M PBS (pH 8). The electrochemical behavior of TPrA has been extensively studied by W. Miao and A. J. Bard, and an ECE′ reaction mechanism, as illustrated in Scheme 1S, has been reported [2]. TPrA is first oxidized at the electrode surface to form the radical cation TPrA^•^⁺ according to eq (3). The heterogeneous electron-transfer rate constant (kₛ) for this E step depends on solution pH and has been reported to be approximately 0.01 cm s⁻¹ at pH 8 [3].


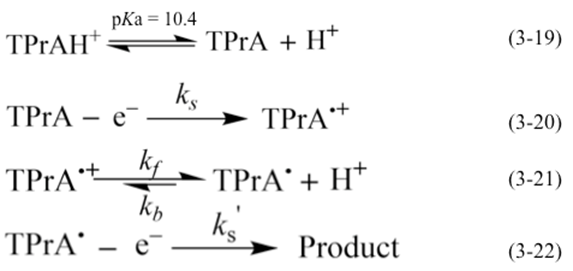


(3)

(4)

(5)

Subsequently, TPrA^•^⁺ undergoes a deprotonation reaction eq (4), yielding the strongly reducing radical TPrA^•^. The forward rate constant (*k*_f_) for the deprotonation reaction has been reported to be approximately 35 s⁻¹[3]. The resulting TPrA^•^ is then further oxidized at the electrode to produce the final oxidation product, and its heterogeneous rate constant is defined as *k*ₛ′ as shown in eq (5).

In the present study, the cyclic voltammogram of TPrA was simulated based on the ECE′ reaction scheme and fitted to the experimental data using a least-squares optimization procedure to refine the thermodynamic and kinetic parameters. The simulated result is shown as black circles in Fig. 3S(A) with the following parameters: *E*°₍_TPrA•⁺/TPrA_₎ = +0.85 V vs. Ag/AgCl; *E*°_TPrA•_ = −1.7 V *vs*. Ag/AgCl; *k*_f_ = 35 s⁻¹; *k*_f_/*k*_b_ (= *K*_a_) = 10⁻⁸; and *k*ₛ = *k*ₛ′ = 0.01 c m s⁻¹. CV simulations were performed using DigiElch Software (Gamry Instruments). The calculated voltammogram agrees well with the experimental data obtained at the glassy carbon electrode, thereby supporting the validity of the proposed ECE′ reaction mechanism for TPrA.


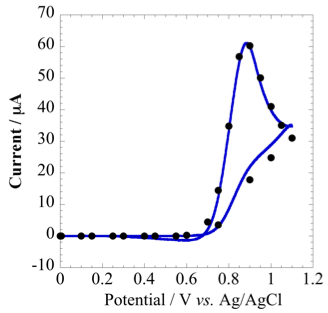
**
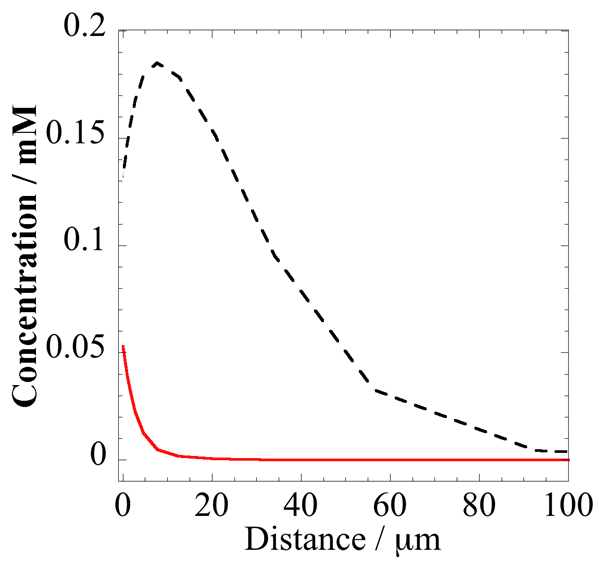
**

**Measured**

**Calculated**

**TPrA^･^**

**TPrA^･+^**

**Fig. 3S.** (A) Cyclic voltammograms of 1 mM TPrA(solid line) at GC electrode in Tris buffer (pH 8.0) and a simulated CV of TPrA oxidation (dotted line) in pH 8.0 solution at a scan rate of 0.1 V/s. Parameters used in the simulation were : *E*^0^_TPrA•⁺_ _/ TPrA_ = 0.85 V vs. Ag/AgCl, *E*^0^_TPrA•_ = − 1.7 V *vs*. Ag/AgCl, *k*_s_ = *k*_s_*’* = 0.01 cm/s, *k*_f_ =3500 s^−1^, *k*_f_ /*k*_b_ =10^−8^, *C*_TPrA_ = 1 mM, All species were assumed to have a diffusion coefficient of 5.0×10^−6^ cm^2^/s except for H^+^ (*D*_H_+ = 5.0×10^—5^ cm^2^/s). (B) The concentration profiles for TPrA^•^⁺ (solid line) and TPrA^•^ (dashed line) at +0.85 V.

Furthermore, using the CV-The Movie function of the simulation software, the concentration profiles of intermediate species at the electrode surface were calculated as a function of potential. As shown in Fig. 3S(B), the profiles of TPrA^•^⁺ and TPrA^•^ near the electrode surface are presented. Notably, around +0.85 V—where the ECL intensity was experimentally observed to be maximal—both TPrA^•^⁺ and TPrA^•^ coexist near the electrode surface in a defined proportion.

**3.** **ECL emission spectrum**

**Fig. 4S**. ECL and PL spectra measured at the surface of a GC electrode in 0.1 M Tris buffer solution (pH 8.5) containing 1 μM CdTe QDs with a diameter of 3.2 nm and 1mM TrPA of CdTe QDs. ECL spectrum was recorded by continuous potential scan between 0 to 1.0 V vs. Ag/AgCl. The potential scan rate was 50 mV/s, and the integration time for the ECL spectrum was 1 min. PL spectrum was measured under the conditions shown in Fig. 1S.

**4.** **Energy level diagram of CdTe QDs with a diameter of 3.2 nm**

In cyclic voltammetry, the oxidation peak appears when electrons are withdrawn from the QDs. In this study, the oxidation onset potential is used as an experimental approximation of the VB energy level (*E*_VB_ = 1.2 V *vs*. Ag/AgCl). The energy level of CB (*E*_VB_) thus corresponds to approximately −0.8 (=1.2-2.0) V *vs*. Ag/AgCl. The energy level diagram of CdTe QDs with a diameter of 3.2 nm and the electron-transfer process associated with fluorescence quenching are schematically illustrated in Fig. 3S. The dashed lines in the figure represent the excited energy level (*E*_s_) of CdTe QDs.


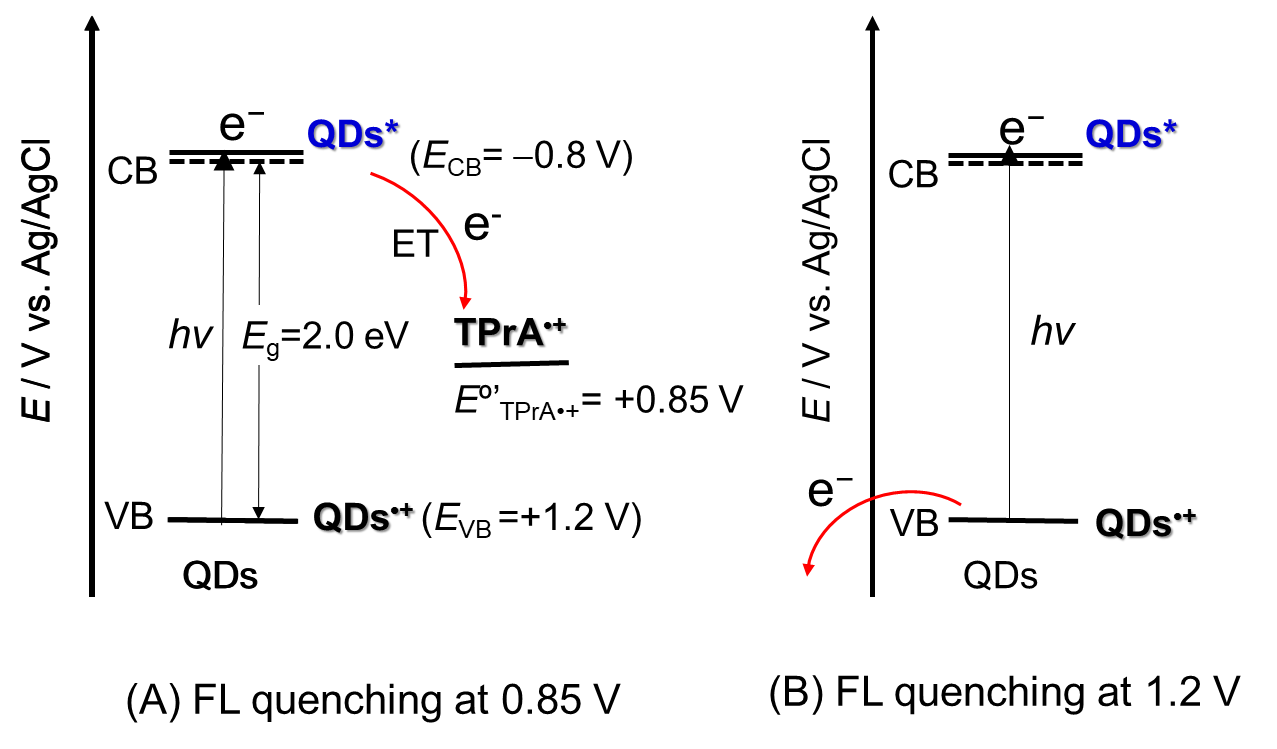


**Fig.5S.** Energy level diagram of CdTe QDs with a diameter of 3.2 nm and the electron-transfer processes associated with fluorescence quenching for CdTe QDs^*^.

**References**

1. W. Yu, L. Qu, W. Guo, and X. Peng, *Chem. Mater.*, 15, 2854 (2003).
2. W. Miao, Jai-Pil Choi, A. J. Bard, J. Am. Chem. Soc., 124, 14478(2002).
3. E. M. Gross, P. Pastore, and R. M. Wightman, J. Phys. Chem. B, 105, 8732 (2001).
